# Asymmetric Whole-Cell Bio-Reductions of (*R*)-Carvone Using Optimized Ene Reductases

**DOI:** 10.3390/molecules24142550

**Published:** 2019-07-12

**Authors:** Christoph Mähler, Christian Burger, Franziska Kratzl, Dirk Weuster-Botz, Kathrin Castiglione

**Affiliations:** 1 Institute of Biochemical Engineering, Technical University of Munich, Boltzmannstr. 15, D-85748 Garching, Germany; 2 Institute of Bioprocess Engineering, Friedrich-Alexander-University Erlangen-Nürnberg, Paul-Gordan-Str. 3, D-91052 Erlangen, Germany

**Keywords:** asymmetric reduction, biotransformation, ene reductase, formate dehydrogenase, (*R*)-carvone, (2*R*,5*R*)-dihydrocarvone

## Abstract

(2*R*,5*R*)-dihydrocarvone is an industrially applied building block that can be synthesized by site-selective and stereo-selective C=C bond bio-reduction of (*R*)-carvone. *Escherichia coli* (*E.* *coli*) cells overexpressing an ene reductase from *Nostoc* sp. PCC7120 (NostocER1) in combination with a cosubstrate regeneration system proved to be very effective biocatalysts for this reaction. However, the industrial applicability of biocatalysts is strongly linked to the catalysts’ activity. Since the cell-internal NADH concentrations are around 20-fold higher than the NADPH concentrations, we produced *E.* *coli* cells where the NADPH-preferring NostocER1 was exchanged with three different NADH-accepting NostocER1 mutants. These *E. coli* whole-cell biocatalysts were used in batch operated stirred-tank reactors on a 0.7 l-scale for the reduction of 300 mM (*R*)-carvone. 287 mM (2*R*,5*R*)-dihydrocarvone were formed within 5 h with a diasteromeric excess of 95.4% and a yield of 95.6%. Thus, the whole-cell biocatalysts were strongly improved by using NADH-accepting enzymes, resulting in an up to 2.1-fold increased initial product formation rate leading to a 1.8-fold increased space-time yield when compared to literature.

## 1. Introduction

Biocatalysts may represent a green and sustainable alternative to chemical catalysts. They can be used under mild conditions and are biodegradable [1]. Due to their high selectivity and specificity, biocatalysis is becoming more the method of choice for the industrial preparation of chiral compounds [2]. Nevertheless, the application of wild type biocatalysts for industrial purposes is often not cost-effective, due to low activities and space-time yields [1,3]. This can be circumvented by the usage of recombinant microorganisms and optimized enzymes [3].

The asymmetric reduction of C=C double bonds is a widely employed reaction, since up to two stereogenic centers can be generated [4]. In this context, ene reductases (ERs) from the old yellow enzyme family (OYE, EC 1.6.99.1) represent highly interesting biocatalysts for *anti*-specific hydrogenations of activated alkenes [5]. Thus, these enzymes have already been applied in industrial-scale biotransformations [5]. However, large-scale production processes with OYEs are cost-intensive and only applicable when products of high value are produced, e.g., active pharmaceutical compounds. One reason for this is that the majority of these oxidoreductases prefer Nicotinamide adenine dinucleotide phosphate (NADPH) over the less expensive and more stable Nicotinamide Adenine Dinucleotide (NADH) as a cosubstrate [6,7,8]. This is not only a drawback if the biocatalysts are used as isolated enzymes and the expensive cosubstrate has to be added externally, but also if the cosubstrate is supplied through the application of whole-cell biocatalysts. The greater intracellular concentration of NAD(H), which is, for instance, up to 20-fold higher than the concentration of NADP(H) in exponentially growing *Escherichia coli* (*E. coli*) cells [9], makes the utilization of this cosubstrate more effective. Additionally, to further reduce process costs, industrial-scale biotransformations necessitating nicotinamide-based cosubstrates are generally conducted with a regeneration system. Thus, an additional advantage of NAD(H)-usage is represented by a larger variety of NAD(H)-regenerating instead of NADP(H)-regenerating systems [10], which facilitates their implementation in industrial processes [11].

Enzyme engineering was applied to reduce the strong NADPH-dependency of OYEs. In a previous work, we were able to influence the cosubstrate preference of the cyanobacterial ene reductase 1 from *Nostoc* sp. PCC7120 (NostocER1) [12]. This was achieved by swapping loop regions that participate in cosubstrate binding. Different regions of NostocER1 were exchanged for the respective regions of two native donor OYEs with a high activity with NADH or a high affinity toward NADH. Thus, the NADH-binding properties of the donors could be transferred to the cyanobacterial ER. This enabled a combination of the very high stereospecifity and broad substrate spectrum of NostocER1 [13] with a high NADH activity. Kinetic parameters of NostocER1 and the three best performing NostocER1-variants are listed in Table 1.

In this study, these optimized enzymes should be applied in a biotransformation of industrial relevance. Therefore, the reduction of (*R*)-carvone to (2*R*,5*R*)-dihydrocarvone was carried out. Dihydrocarvone is used in the synthesis of different molecules of biological interest, e.g., tetraoxane derivatives as compounds with antimalarial activity [14], keto decalin derivatives as insect antifeedants [15], or the terpenes (−)-thujopsene [16] and (+)-decipienin A [17]. Due to the reduced appearance of undesired side products during the reduction of (*R*)-carvone, especially when compared to Baker’s yeast [18,19], recombinant *E. coli* biocatalysts overexpressing optimized NostocER1 mutants were applied.

## 2. Results

### 2.1. Strain Construction

**Figure 1:**
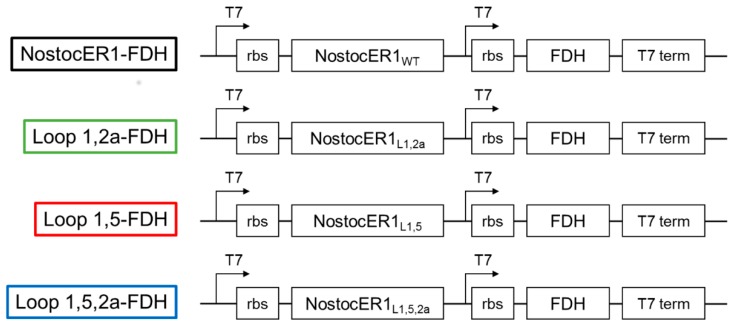
Modified pET28a(+) vector constructs for the co-expression of the respective *NostocER1* variants and *FDH* genes. Indicated are the expression cassettes, including the locations of the T7 promotors (T7), ribosome binding sites (rbs), the genes (NostocER1 and FDH), and the T7 terminators (t7 term).

First different vectors for the transformation of *E. coli* BL21(DE3) cells were constructed (Figure 1). These vectors are based on a pET28a(+) vector, with the exception of the expression cassettes. An additional T7 promotor as well as a ribosome binding site (rbs) were inserted for an improved co-expression of the genes encoding for NostocER1 and the formate dehydrogenase (FDH) from *Mycobacterium vaccae*, which was used for cosubstrate regeneration [19]. Four different NostocER1 variants were applied, i.e., the wild type, a loop 1,2a swapped mutant with a highly increased activity with NADH, a loop 1,5 swapped mutant with a highly increased affinity toward NADH, and the combination of these two, a loop 1,5,2a swapped mutant with an increased activity and affinity with NADH. Details of differences in the primary structure and kinetic parameters can be found in a previous work [12] and Table 1.

### 2.2. Biocatalyst Production

**Figure 2:**
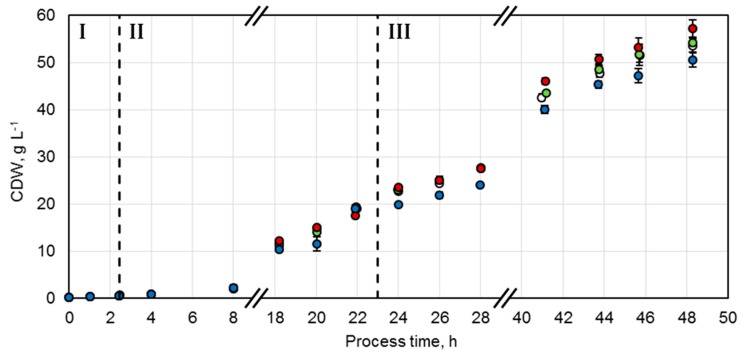
Cell dry weight (CDW) concentrations of fed-batch cultivations of *E. coli* BL21 (DE3) with constructs NostocER1-FDH (○), Loop 1,2a-FDH (●), Loop 1,5-FDH (●), and Loop 1,5,2a-FDH (●) in a stirred-tank bioreactor on an L-scale. The vertical dotted lines separate the biomass production in the batch phase (I), the fed-batch phase (II), and the enzyme production phase (III). All values represent means ± standard deviation of triplicate determinations.

A protocol adjusted from Wilms et al. was established for the production of the whole-cell biocatalysts [20]. Studies showed that, for *E. coli*, growth rates should be maintained between 0.1 h^−1^ and 0.3 h^−1^ during the biomass production phase in order to reduce overflow metabolism, which leads to inhibitory concentrations of acetate [21]. Control of the growth rate can be achieved by a fed-batch process with an exponential feeding strategy of the limiting substrate. Consequently, after an initial batch phase (Phase I) with 1 g L^−1^ glucose at 37 °C, an exponential feeding profile of glucose was initiated in order to maintain a specific growth rate of µ_set_ = 0.15 h^−1^ at 30 °C for biocatalyst production (Phase II). This was followed by an enzyme production phase (Phase III), which was induced by 200 µM isopropyl β-d-1-thiogalactopyranoside (IPTG) after 23.1 h. The exponential feeding protocol was adjusted to a reduced specific growth rate of µ_set_ = 0.04 h^−1^ at an initial temperature of 20 °C. With this strategy, four parallel fed-batch processes were conducted to achieve identical biocatalyst production conditions. Thereby, a comparable course and maximum of the biocatalyst concentrations for each variant could be accomplished (Figure 2). The initial glucose concentration of 1 g L^−1^ was consumed after 2.5 h (Phase I) and it was possible to control the specific growth rates between 0.139 ± 0.001 h^−1^ and 0.155 ± 0.006 h^−1^ by exponential glucose feeding (Phase II). During the enzyme production phase (Phase III), the specific growth rates were successfully maintained between 0.035 ± 0.001 h^−1^ and 0.038 ± 0.001 h^−1^ by exponential glucose feeding. Since the additional energy required for protein production can influence the biomass yield coefficient, an increased substrate supply was set at the beginning of this phase. Since no change of the coefficient was observable, glucose was overfed and accumulated up to 2.4 g L^−1^. Thus, to enhance the growth metabolism, the temperature was increased from 20 to 23 °C after 30.5 h of process time. Hereby, glucose concentrations could be kept below 0.2 g L^−1^.

A maximal cell dry weight (CDW) concentration of 53.5 ± 1.6 g L^−1^ was achieved after 48.3 h with the *E. coli* strain overexpressing the wild type enzymes (NostocER1-FDH). The constructs with the two NostocER1-variants Loop 1,2a-FDH and Loop 1,5-FDH showed comparable maximal CDW concentrations after 48.3 h (54.2 ± 1.1 g L^−1^ and 57.1 ± 2.0 g L^−1^, respectively). The lowest final CDW concentration of 50.6 ± 1.6 g L^−1^ was observed with the NostocER1-variant Loop 1,5,2a-FDH. This was, however, due to an oxygen limitation because of a blocked waste-gas filter of the stirred-tank bioreactor between 12.3 h and 16.3 h of process time.

**Figure 3:**
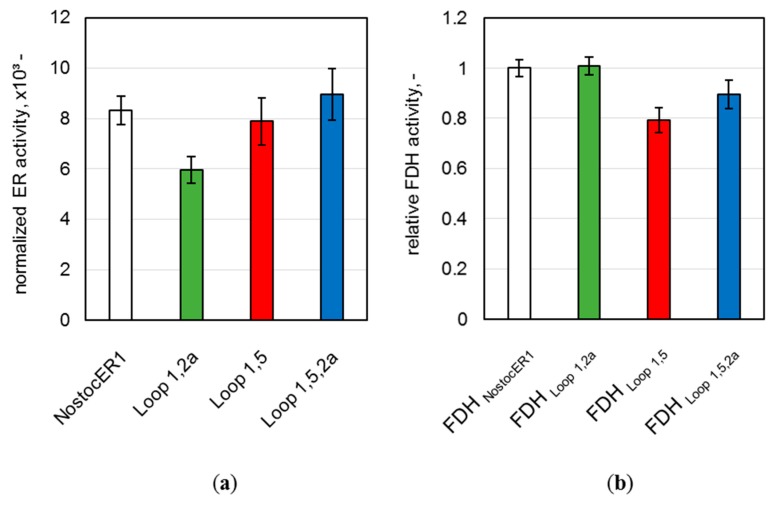
Activities in the supernatant of the disrupted *E. coli* cells after a process time of 48.3 h. (**a**) Ene reductase (ER) activity normalized to the activity of respective purified NostocER1-variant. Activities were determined by measuring the oxidation rate of 500 µM NADH. (**b**) Formate dehydrogenase (FDH) activities of the *E. coli* cells with optimized NostocER1-variants relative to the FDH activity of the *E. coli* cells possessing wild type enzymes (NostocER1-FDH construct). Activities were determined by measuring the reduction rate of 500 µM NAD^+^.

To further evaluate possible differences of the whole-cell biocatalysts produced, the final enzymatic activities of all NostocER1-variants and all FDHs were determined. A comparison of these activities allows a direct comparison of the intracellular enzyme amount. Therefore, part of the harvested *E. coli* cells were mechanically disrupted and the enzymatic activities were determined in the supernatant. Since the four applied NostocER1-variants possess different activities with NADH [12], a direct comparison of the specific activities is not possible. Therefore, the determined activities were normalized to the specific activity using 500 µM NADH (k_500_) of the respective purified NostocER1-variant (Table 1). All optimized NostocER1-variants revealed an insignificantly changed (Loop 1,5 and Loop 1,5,2a) or significant lower (Loop 1,2a) normalized activity compared to the NostocER1 wild type (Figure 3a). Thus, higher enzyme amounts of the optimized ERs can be excluded as reason for different biotransformation rates compared to the *E. coli* biocatalyst possessing the wild types (NostocER1-FDH). In order to ensure the same for the cosubstrate regeneration activity, the supernatant activities of the FDHs were determined and compared to one of the cells possessing the wild type enzymes. Since all constructs hold the gene encoding for the same FDH, a direct comparison of the FDH activities was possible (Figure 3b). Again, none of the FDHs of the *E. coli* cells with a construct holding an optimized NostocER1 revealed a significantly higher activity than the ones with the construct with the wild type NostocER1, which indicates comparable expression levels. Hence, the biotransformation rates of the whole-cell biocatalysts possessing optimized NostocER1-variants are directly comparable to the cells possessing the wild types.

### 2.3. Biotransformation of (R)-Carvone

**Figure 4:**
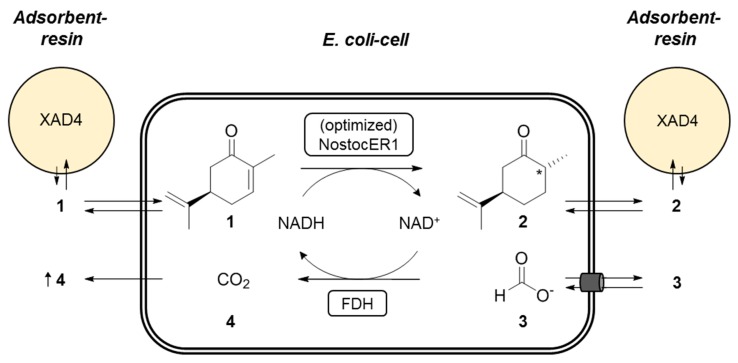
Schematic representation of the applied biocatalytic whole-cell reduction of (*R*)-carvone (**1**) to (2*R*,5*R*)-dihydrocarvone (**2**). During this NostocER1 catalyzed reaction, intracellular NADH is consumed. Therefore, NAD^+^ gets recycled inside the cells via a formate dehydrogenase (FDH) under the oxidation of formate (**3**) to carbon dioxide (**4**). The adsorbent resin (AR) Amberlite^®^ XAD4 is used for in situ substrate feeding and product removal (SFPR).

The recombinant *E. coli* cells were used in whole-cell biotransformations of (*R*)-carvone (**1**) to form (2*R*,5*R*)-dihydrocarvone (**2**). Intracellular NADH is consumed by the NostocER1 wild type or the three optimized enzyme variants. The formed NAD^+^ is recycled inside the whole-cell biocatalysts via the formate dehydrogenase (FDH) from *Mycobacterium vaccae* by oxidation of formate (**3**) to carbon dioxide (**4**). Since **1** is poorly soluble in aqueous media and shows antimicrobial activity, the Amberlite^®^ XAD4 resin was added for in situ substrate feeding and product removal (SFPR). This resin resulted in beneficial results compared to other SFPR strategies, i.e., different organic solvents or ionic liquids [19]. A schematic representation of the reaction setup applied is shown in Figure 4.

First, this reaction setup was applied in stirred small-scale batch biotransformations to evaluate technical replicability. Second, the evaluation of preparative biotransformations with all biocatalysts was performed on a 0.7 L scale in stirred-tank bioreactors.

#### 2.3.1. Technical Replicability

**Figure 5:**
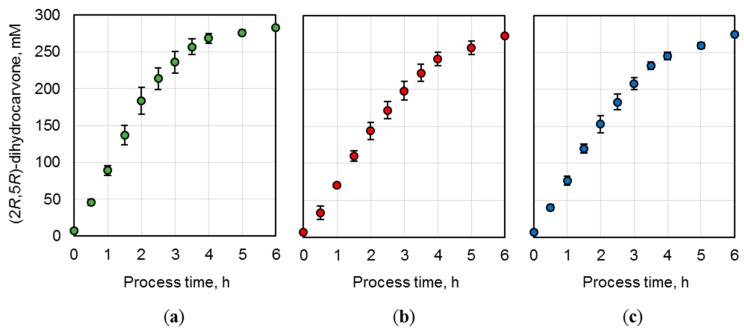
(2*R*,5*R*)-dihydrocarvone concentration over process time during batch biotransformation on a 15 mL scale. *E. coli* BL21 (DE3) whole-cell biocatalysts were applied after expression of (**a**) Loop 1,2a-FDH construct, (**b**) Loop 1,5-FDH construct, and (**c**) Loop 1,5,2a-FDH construct. The reactions were performed with 300 mM (*R*)-carvone, 450 mM formate, a biocatalyst cell dry weight (CDW) concentration of (**a**) 40.2 g_CDW_ L^−1^, (**b**) 51.3 g_CDW_ L^−1^, and (**c**) 39.6 g_CDW_ L^−1^. A resin to substrate ratio of 3:1 in sodium phosphate buffer (0.3 M, pH 7.0) at 25 °C. The values are means of three technical replicates ± standard deviations.

To ensure a technically replicable application of the produced whole-cell biocatalysts, biotransformations of (*R*)-carvone were conducted on a 15 mL scale in triplicates. The *E. coli* BL21 (DE3) cells possessing optimized NostocER1-variants (i.e., the vectors Loop 1,2a-FDH, Loop 1,5-FDH, and Loop 1,5,2a-FDH) were applied in three parallel batch biotransformations per construct (Figure 5). The means and standard deviations of these three replicates were calculated. Thereby, a maximal relative standard deviation of ±9.9% and average relative standard deviations of ±5.7% were determined for the Loop 1,2a-FDH construct (a), ±5.2% for the Loop 1,5-FDH construct (b), or ±5.6% for the Loop 1,5,2a-FDH construct (c).

To compare the biocatalysts’ activity, the initial product formation rates were determined per biocatalyst amount. Loop 1,2a-FDH construct (a) revealed the highest rate with an initial (2*R*,5*R*)-dihydrocarvone formation rate of 2.2 ± 0.2 mmol h^−1^ g_CDW_^−1^. The rates of the Loop 1,5-FDH construct (b) (1.4 ± 0.1 mmol h^−1^ g_CDW_^−1^) and the Loop 1,5,2a-FDH construct (c) (1.9 ± 0.1 mmol h^−1^ g_CDW_^−1^) were reduced by 38.0 ± 5.3% and 14.9 ± 1.6%, respectively.

#### 2.3.2. Preparative Biotransformations

**Figure 6:**
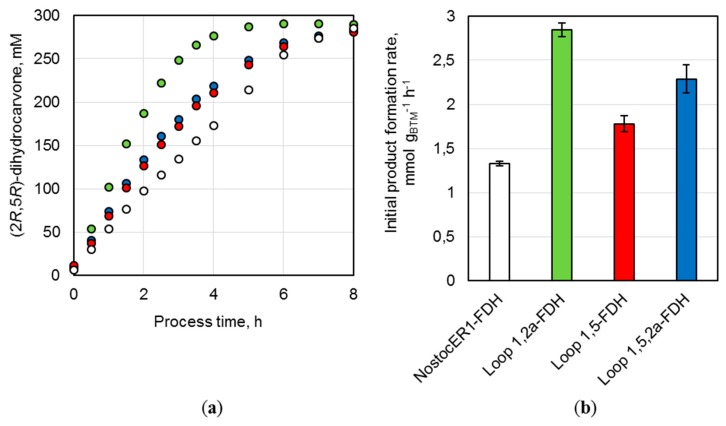
Batch biotransformations in stirred-tank bioreactors on a 0.7 L scale using the recombinant *E. coli* BL21 (DE3) whole-cell biocatalysts NostocER1-FDH (white), Loop 1,2a-FDH (green), Loop 1,5-FDH (red), and Loop 1,5,2a-FDH (blue). The reactions were performed with 300 mM (*R*)-carvon, 450 mM formate, 0.1 mM NAD^+^, a biocatalyst cell dry weight (CDW) concentration of 30.5–33.7 g_CDW_ L^−1^, and a resin to substrate ratio of 3:1 in sodium phosphate buffer (0.3 M, pH 7.0) at 25 °C. (**a**) (2*R*,5*R*)-dihydrocarvone concentration over process time. (**b**) Initial (2*R*,5*R*)-dihydrocarvone formation rates of the applied biocatalysts per biomass. All values represent means ± standard deviation of triplicate determinations.

Parallel batch processes were performed with three recombinant *E. coli* strains possessing an optimized NosctocER1-variant and the strain with the NostocER1 wild type in stirred-tank bioreactors on a 0.7 L scale. All whole-cell biocatalysts led to an almost complete conversion of (*R*)-carvone to (2*R*,5*R*)-dihydrocarvone within 8 h of a reaction time (Figure 6a). Thereby, the usage of all *E. coli* cells possessing an optimized NostocER1-variant resulted in a faster reaction rate. While the biocatalysts with the wild type (NostocER1-FDH) showed a product concentration of 97.9 ± 1.5 mM after 2 h process time, the other three *E. coli* strains ranged between 187.0 ± 1.4 mM (Loop 1,2a-FDH) and 126.6 ± 0.8 mM (Loop 1,5-FDH). The same differences were observed with respect to the initial (2*R*,5*R*)-dihydrocarvone formation rates per applied biomass (Figure 6b). *E. coli* cells with the NostocER1 wild type possessed the slowest of all determined product formation rates (1.3 ± 0.1 mmol h^−1^ g_CDW_^−1^). The application of the Loop 1,2a-FDH construct increased the product formation rate by a factor of 2.1 to 2.8 ± 0.1 mmol h^−1^ g_CDW_^−1^. The other two biocatalysts also showed an increased product formation rate. The Loop 1,5-FDH construct resulted in an increase by a factor of 1.3 to 1.8 ± 0.1 mmol h^−1^ g_CDW_^−1^ and the Loop 1,5,2a-FDH construct in an increase by a factor of 1.7 to 2.3 ± 0.2 mmol h^−1^ g_CDW_^−1^. A comparable stereospecificity of all biocatalysts could be observed, which results in similar diastereomeric excesses (de) of the product (2*R*,5*R*)-dihydrocarvone compared to the side product (2*S*,5*R*)-dihydrocarvone. The whole-cell biocatalysts with NostocER1-FDH and the Loop 1,2a-FDH revealed the highest stereospecificity after ≥96% conversion with a de of 95.4%. The corresponding de of Loop 1,5-FDH (93.6%) and Loop 1,5,2a-FDH (94.3%) were slightly lower.

## 3. Discussion

In this study, (*R*)-carvone was successfully reduced to (2*R*,5*R*)-dihydrocarvone using recombinant *E. coli* whole-cell biocatalysts overexpressing *nostocER1* variants and *fdh* genes. The application of bacterial whole-cell biocatalysts for this reduction reaction was already demonstrated to be beneficial in comparison to other microorganisms, since conversions of ≥96% were reached whereas the undesired reduction of carbonyl bonds by endogenous carbonyl reductases was not observed [19,22,23]. The reduction of the C=O bonds of substrate or product was mostly an issue when applying other organisms like yeasts [18,24,25] and other fungi [24,26], plant cells [27], or microalgae [28].

In a previous work, the overexpression of the NADPH-preferring cyanobacterial ene reductase from *Nostoc* sp. PCC7120 (NostocER1) in combination with a NADP^+^-accepting mutant of formate dehydrogenase (FDH_3M_) from *Mycobacterium vaccae* resulted in highly active *E. coli* whole-cell biocatalysts [19]. 300 mM (*R*)-carvone were converted to 290.4 mM (2*R*,5*R*)-dihydrocarvone in a batch process on a liter-scale after 9 h by applying a biocatalyst concentration of 36 g_CDW_ L^−1^. A space-time yield of 32.3 mmol L^−1^ h^−1^ was achieved with an initial (2*R*,5*R*)-dihydrocarvone formation rate of 1.3 mmol h^−1^ g_CDW_^−1^. In this study, the NostocER1 wild type was overexpressed in combination with the NAD^+^-preferring FDH wild type. Thereby, *E. coli* biocatalysts could be provided showing a similar activity compared to literature. On a 0.7 L scale 285.4 mM (2*R*,5*R*)-dihydrocarvone were formed in a batch process within 8 h with an initial product formation rate of 1.3 ± 0.1 mmol h^−1^ g_CDW_^−1^, which results in a space-time yield of 35.7 mmol L^−1^ h^−1^. Thus, within this process time, a yield of 95.1% was achieved based on 210 mmol applied substrate (*R*)-carvone. However, further improved biocatalysts were generated by exchanging the NostocER1 wild type with different NADH-accepting NostocER1-variants (Table 2). The implementation of the Loop 1,2a NostocER1-variant, which possesses a highly increased catalytic constant with NADH [12], resulted in significant improvement. Thereby, a yield of 95.7% was achieved after a process time of 5 h. The initial product formation rate was increased 2.1-fold to 2.8 ± 0.1 mmol h^−1^ g_CDW_^−1^ and the space-time yield after ≥96% conversion was increased 1.8-fold to 57.4 ± 0.1 mmol L^−1^ h^−1^ when compared to literature [19]. This was achieved even though the expression level of this ER was between 24.3 ± 3.6% and 33.4 ± 4.9% lower than the levels of the other biocatalysts. Additionally, the application of the two other NostocER1-variants (Loop 1,5 resp. Loop 1,5,2a) led to higher initial (2*R*,5*R*)-dihydrocarvone formation rates. However, no further improved space-time yields were observed with these two variants in comparison to the NostocER1 wild type biocatalysts.

The bio-reduction of (*R*)-carvone has already been studied using many different microorganisms, including bacteria [19,23], yeasts [18,24,25], other fungi [24,26], plant cells [27], or microalgae [28]. With more than 30 different strains, yeasts and yeast-like fungi are the largest group studied for this reaction [18,24,25]. High substrate conversions of ≥96%, which are a crucial requirement for a cost-effective industrial biotransformation, were only achieved with one strain, i.e., *Hanseniaspora guilliermondii* [24]. However, only a moderate substrate concentration of 6.9 mM was converted. A significantly reduced conversion (63.6%) was detected after the substrate concentration was increased to 10 mM for the bio-reduction with this organism [25]. Through the application of the yeast *Saccharomyces cerevisiae* and the addition of trehalose to the reaction mixture, the substrate loading could be increased to 16.6 mM and a conversion of 74% was reached [18].

Besides yeasts, bacteria proved to be beneficial for the selective reduction of (*R*)-carvone. In this context, *Pseudomonas putida* was used to fully convert 6.9 mM of the substrate with a high stereospecificity within 84 h [23]. A strong improvement was achieved by the application of *E. coli* cells overexpressing the ene reductase NostocER1 and a FDH for cosubstrate regeneration. The high effectiveness of this biocatalyst was demonstrated in a biphasic system with the adsorbent resin XAD4, which results in the, by far, highest product formation rate and space-time yield for the bio-reduction of (*R*)-carvone in literature [19]. Furthermore, a high and stereospecific conversion of 96.8% of the substrate was reached within 9 h of processing time [19]. In this study, this biocatalyst was further improved by applying NADH-accepting variants of NostocER1 [12], which results in up to 99.4% conversion of 300 mM (*R*)-carvone within 6 h of process time. The product formation rate was increased by a factor of up to 2.1 and the space-time yield by a factor of 1.8. Thereby, the Loop 1,2a NostocER1-variant, i.e., the variant with the highest k_cat_ with NADH of the three applied NostocER1-variants (Table 1), revealed the biggest improvement. This is indicative of a stronger influence of k_cat_ with NADH compared to K_m_ towards NADH on the productivity of the biocatalyst in the given reaction system. This observation is in accordance with the high NAD(H) concentration in *E. coli*, which is up to 20-fold higher compared to NADP(H) in exponentially growing cells [9]. Through a high cosubstrate concentration, the apparent activity is closer to the maximal turnover number (k_cat_), which increases the importance of this parameter.

Another important aspect of this study is that we could not only improve the bioreduction of (*R*)-carvone, but also demonstrate that cosubstrate engineering of an NADPH-dependent oxidoreductase is useful, even if whole-cell biocatalysts are applied.

## 4. Materials and Methods

### 4.1. Chemicals, Enzymes, and Bacterial Strains

(*R*)-carvone (98%), dihydrocarvone (mixture of isomers 77% (2*R*,5*R*), and 20% (2*S*,5*R*)) and Amberlite^®^ XAD4 were obtained from Sigma Aldrich (St. Louis, MO, USA). Media components, Kanamycin sulfate, and NAD(H) were purchased from Carl Roth (Karlsruhe, Germany). Enzymes for DNA manipulation were bought from New England Biolabs (Ipswich, MA, USA) and primers were synthesized by Eurofins Genomics (Ebersberg, Germany). *E. coli* DH5α (Invitrogen, Carlsbad, CA, USA) was used for cloning and *E. coli* BL21 (DE3) (Novagen, San Diego, CA, USA) for protein expression and biotransformation.

### 4.2. Cloning

Standard procedures were used for polymerase chain reaction, ligation, transformation, and plasmid and cell preparation as described by Mülhart [29]. The constructs for co-expression of the NostocER1-variants and FDH based on a pET28a(+) vector (Novagen, San Diego, CA, USA). The vectors expression cassettes were modified with an additional T7 promotor and ribosome binding site (rbs) as described before [19]. Genes encoding for the NostocER1-variants [12] and the *fdh* gene from *Mycobacterium vaccae* (GenBank BAB69476.1) were amplified using the primers listed in Table 3. For amplification Phusion^®^ High-Fidelity DNA Polymerase (New England Biolabs, Ipswich, MA, USA) was applied according to the manufacturer’s protocol. For *nostocER1* and all variants of this gene, the restriction sites NcoI and BamHI were used. Additionally, *fdh* NdeI and KpnI was applied.

### 4.3. Cultivation of E. coli

Biocatalyst production in l-scale was performed in four-fold parallel lab-scale stirred-tank reactors with an initial volume of 0.3 L (DASGIP^®^ Bioblock, DASGIP^®^ reactor with two GL45 sides and overhead propulsion, Eppendorf AG, Hamburg, Germany). Temperature, dissolved oxygen concentration (DO), pH, and gas flow rate were monitored during the production of the whole-cell biocatalysts. The temperature was initially set to 37 °C for the batch phase, which was reduced to 30 °C for the fed-batch phase and was further reduced to 20–23 °C after induction for the enzyme production phase. The pH was controlled to pH 7.0 by 12.5% (*v*/*v*) ammonia and 1 M phosphoric acid addition. Solved oxygen availability was achieved in the reactors by the supply of sterile air with a sparger at the bottom and dispersion of the gas bubbles with two six-blade Rushton turbines mounted on the axes at a distance of 25 mm. The DO concentration was controlled to a set point of 20% air saturation by varying the stirrer speed between 500 and 1600 rpm. The air flow was set to 60 L h^−1^ representing an air flow of at least 3.33 volume per volume of the vessel capacity per minute (vvm). DO sensor calibration was performed prior to inoculation and 100% air saturation was achieved by stripping with air at 37 °C. Afterward, the reactors were stripped with nitrogen gas until 0% air saturation was achieved.

Pre-cultures were produced with *terrific broth* (TB) medium containing 12 g L^−1^ peptone from casein, 24 g L^−1^ yeast extract, 5.1 g L^−1^ glycerol, 2.1 g L^−1^ KH_2_PO_4_, 12.6 g L^−1^ K_2_HPO_4_, and 34 mg L^−1^ kanamycin. The pH was adjusted to pH 7.2 and 4 mL of the TB medium were inoculated either with a single cell colony or cells from a cryo-stock in a 13 mL single use cultivation tube (Sarstedt, Nümbrecht, Germany). Seed cultures and biocatalyst production in stirred-tank bioreactors were carried out in defined medium, according to Wilms et al. [20]. Seed cultures with an initial glucose concentration of 5 g L^−1^ were grown overnight at 37 °C in shaking flasks without baffles (250 rpm, shaking diameter of 5 cm). Cells from the seed cultures were harvested by centrifugation (3250× *g*, 10 min) and the required amount of the cell pellet was resuspended in fresh medium, according to Wilms et al. Afterward the re-suspended cells were used for inoculation of the stirred-tank bioreactors by sterile single-use syringes through a cannula.

Glucose served as a substrate for the initial batch phase at a concentration of 1 g L^−1^. Kanamycin was used as a selection marker at a concentration of 34 mg L^−1^. For fed-batch cultivations, a growth rate of µ_set_ = 0.15 h^−1^ was set by using a biomass yield coefficient of 0.4 g_CDW_ g_S_^−1^ and, adding a feeding solution with 100 g L^−1^ glucose, 19.8 g L^−1^ (NH_4_)_2_HPO_4_ and 34 mg L^−1^ kanamycin, according to Sun et al. [30]. Temperature was reduced to 20 °C after a process time of 19.5 h and 1 h prior to the induction of enzyme production with 200 µM IPTG. 7.5 h after induction, the temperature was raised to 23 °C due to glucose accumulation in the stirred-tank reactor. During the production phase, the growth rate was reduced to µ_set_ = 0.04 h^−1^ and a feeding solution with 500 g L^−1^ glucose, 99 g L^−1^ (NH_4_)_2_HPO_4_, and 34 mg L^−1^ kanamycin was used. Furthermore, 0.5 g L^−1^ MgSO_4_ · 7 H_2_O and 3 mL L^−1^ trace element solution [20] were added to the stirred-tank reactors after 5.5 and 24.5 h. At 24.5 h, an additional 0.1 g L^−1^ thiamin was supplemented to the reaction medium. Cells were transferred to centrifugation tubes after cell cultivation and were stored for less than 24 h at 4 °C until further use for preparative biotransformations. The cells for the analysis of the technical replicability were mixed with 12.5% (*w*/*v*) glycerol and stored at −60 °C.

### 4.4. Enzyme Activity Assay

Enzyme activities were determined in cell lysates. Therefore, 1 mL *E. coli* cell suspension was mixed with 0.5 g glass beads (ø 0.25–0.5 mm, Carl Roth, Karlsruhe, Germany) and disrupted for 5 min and 25 s^−1^ in a Mixer Mill MM 400 (Retsch, Haan, Germany). Debris was separated by centrifugation (17,200× *g*, 10 min, 4 °C) and the activity in the supernatant was determined photometrically by oxidation (ER) or reduction (FDH) of 500 µM NAD(H) at 340 nm. Assays were conducted in sodium phosphate buffer (100 mM, pH 7.0) at 30 °C using F96 microwell plates (Nunc, Roskilde, Denmark) and a Multiscan™ FC Photometer (Thermo Fisher Scientific, Waltham, MA, USA). Reactions were initiated by the addition of 10 mM maleimide (ER) or 250 mM sodium formate (FDH) as a substrate. Reaction rates were determined by automated linear regression using MATLAB R2015b (The MathWorks, Natick, MA, USA).

### 4.5. Biotransformations of (R)-Carvone

Biotransformations of (*R*)-carvone were carried out on two different scales: 15 mL for the evaluation of technical replicability and 0.7 L for the preparative biotransformation in the stirred-tank bioreactors. All biotransformations were performed with 300 mM (*R*)-carvone and 450 mM sodium formate in 300 mM sodium phosphate buffer (pH 7.0). Amberlite^®^ XAD4 with a wet mass ratio of 3:1 to *(R*)-carvone was used for in situ substrate feeding and product removal. (*R*)-carvone and adsorbent resin (harmonic mean size 0.49–0.69 mm, uniformity coefficient ≤2) were incubated for at least 2 h in buffer (50% (*v*/*v*) of the total reaction volume) prior to the biotransformations. Reactions were initiated by the addition of the whole-cell biocatalysts resuspended in 300 mM sodium phosphate buffer and the samples were incubated at 25 °C.

On the 15 mL scale, batch biotransformations were performed in glass vials with rolled rim equipped with snap caps (V_max_ 20 mL, Carl Roth, Karlsruhe, Germany). Mixing was achieved with x-shaped magnetic stir bars (ø 1 cm, VWR, Darmstadt, Germany) using a multiple stirrer plate Variomag Poly 15 (HP-Labortechnik, Oberschleißheim, Germany) at 900 rpm. Sampling was done with a volume of 300 µL each.

On the 0.7 L scale, batch biotransformations were performed in parallel stirred-tank reactors (DASGIP^®^ Bioblock, DASGIP^®^ reactor with two GL45 sides and overhead propulsion, Eppendorf, Hamburg, Germany). Furthermore, 100 µM NAD^+^ were added to the reaction mixture. Mixing and dispersion of the gas phase was achieved with two six-bladed Rushton turbines mounted on the axes at a distance of 25 mm and a speed of 700 rpm. The pH was controlled to pH 7.0 by automated addition of 1 M phosphoric acid. Sampling was done with a volume of 1 mL each.

### 4.6. Analytics

Prior to the analytical procedure, the samples had to be extracted using EtOAc. Therefore, between 33% and 50% (*v*/*v*), EtOAc was added to the aqueous-adsorbent resin samples and extraction was performed for 15 min at 25 s^−1^ in a Mixer Mill MM 400 (Retsch, Haan, Germany) for enhanced mass transfer. The phases were separated by centrifugation (17,200× *g*, 5 min) and 100 µL of the organic phase was dissolved in 300 µL EtOAc and 100 µL (*R*)-limonene (36 mM in EtOAc) as an internal standard.

Analytics were performed using a gas chromatograph GC-2010 Plus (Shimadzu, Kyōto, Japan) equipped with a flame ionization detector (FID) and a chiral Lipodex-E column (length 25 m, ø 0.25 mm, Macherey-Nagel, Düren, Germany). The injection volume was set to 1 µL and the split ratio was 10. The injector temperature was set to 200 °C and the detector temperature was set to 250 °C. All runs were performed with the same oven temperature program, i.e., 60 °C for 3 min followed by 3 °C min^−1^ up to 120 °C. Typical retention times were: (*R*)-limonene 7.5 min, (2*R*,5*R*)-DHC 17.4 min, (2*S*,5*R*)-DHC 18.8 min, and (*R*)-carvone 20.2 min. Compound concentrations were determined using a standard from 0.1 mM to 30 mM of the reference compounds. The application of a biphasic system led to high fluctuations of the determined concentrations. Therefore, substrates and products were summarized and normalized to the applied total concentration of 300 mM. Diastereomeric excesses (de) were determined by comparison of the (2*R*,5*R*)-dihydrocarvone concentration with the (2*S*,5*R*)-dihydrocarvone concentration. Corresponding standard deviations of three technical replicates were between 0.01% and 0.04%. Initial (2*R*,5*R*)-dihydrocarvone formation rates were determined by linear regression until a product concentration of 150 mM was achieved.

## Figures and Tables

**Table 1:** Kinetic parameters of the NostocER1 wildtype and three NostocER1-variants with altered NADH-binding properties as published by Mähler et al. [12]. Listed are the catalytic constant (k_cat_), Michaelis-Menten constant (K_m_), and the resulting catalytic efficiency (k_eff_) with NADH, as well as the specific activity using 500 µM NADH (k_500_).

Enzyme	k_cat_, s^−1^	K_m_, µM	k_eff_, s^−1^ mM^−1^	k_500_, s^−1^
NostocER1	15.2 ± 1.9	1050 ± 220	14.4 ± 3.5	3.9 ± 0.2
Loop 1,2a	29.1 ± 0.4	224 ± 11	130.0 ± 6.7	19.2 ± 1.3
Loop 1,5	8.6 ± 0.9	73 ± 13	119.0 ± 25.3	7.5 ± 0.9
Loop 1,5,2a	19.0 ± 0.4	161 ± 11	118.4 ± 8.7	13.3 ± 1.5

**Table 2:** Comparison of the generated biocatalysts.

Biocatalyst	Final Conversion, %	Initial Product Formation Rate, mmol h^−1^ g_CDW_^−1^	Space-Time Yield ^2^, mmol L^−1^ h^−1^
NostocER1-FDH	99.9	1.3 ± 0.1	35.7 ± 0.1
Loop 1,2a-FDH	99.9	2.8 ± 0.1	57.4 ± 0.1
Loop 1,5-FDH	99.6	1.8 ± 0.1	35.1 ± 0.1
Loop 1,5,2a-FDH	99.5	2.3 ± 0.2	35.4 ± 0.1
NostocER1-FDH_3M_^1^	96.8	1.3 ± 0.1	32.3 ± 0.1

^1^ Data from Reference [19]; ^2^ Space-time yields were determined after ≥96% conversion.

**Table 3:** Primers used for amplification of the *nostocER1* and *fdh* genes. The 5′ **→** 3′ sequences with the restriction site shown in bold and the start/stop codon underlined are listed.

Primer	Sequence, 5′ → 3′
nostocER1-NcoI-for	GGAATTC**CCATGG***GC*TCTACCAACATCAACCTATTCTCT
nostocER1-BamHI-rev	CGC**GGATCC**TTACTTATTAGCAACTGCTAAAAATGG
fdh-NdeI-for	AGGAATTC**CATATG**GCAAAGGTCCTGTGCGT
fdh-KpnI-rev	CGG**GGTACC**TCAGACCGCCTTCTTGAACTTGG
